# The effect of exercise and beta_2_-adrenergic stimulation on glutathionylation and function of the Na,K-ATPase in human skeletal muscle

**DOI:** 10.14814/phy2.12515

**Published:** 2015-08-21

**Authors:** Carsten Juel, Morten Hostrup, Jens Bangsbo

**Affiliations:** 1 Department of Biology, University of CopenhagenCopenhagen, Denmark; 2 Department of Nutrition, Exercise and Sports, University of CopenhagenCopenhagen, Denmark

**Keywords:** Na,K-pump, oxidative stress, skeletal muscle membranes

## Abstract

Potassium and sodium displacements across the skeletal muscle membrane during exercise may cause fatigue and are in part controlled by the Na,K-ATPase. Regulation of the Na,K-ATPase is therefore important for muscle functioning. We investigated the effect of oxidative stress (glutathionylation) on Na,K-ATPase activity. Ten male subjects performed three bouts of 4-min submaximal exercise followed by intense exercise to exhaustion with and without beta_2_-adrenergic stimulation with terbutaline. Muscle biopsies were obtained from m. vastus lateralis at rest (Control samples) and at exhaustion. In vitro glutathionylation reduced (*P* < 0.05) maximal Na,K-ATPase activity in a dose-dependent manner. Na,K-ATPase *α* subunits, purified by immunoprecipitation and tested by glutathione (GSH) antibodies, had a basal glutathionylation in Control samples and no further glutathionylation with exercise and beta_2_-adrenergic stimulation. Immunoprecipitation with an anti-GSH antibody and subsequent immunodetection with *β*1 antibodies showed approximately 20% glutathionylation in Control samples and further glutathionylation after exercise (to 32%) and beta_2_-adrenergic stimulation (to 38%, *P* < 0.05). Combining exercise and beta_2_-adrenergic stimulation raised the *β*1 glutathionylation to 45% (*P* < 0.05). In conclusion, both *α* and *β*1 subunits of the Na,K-ATPase were glutathionylated in Control samples, which indicates that the maximal Na,K-ATPase activity is overestimated if based on protein density only. *β*1 subunits are further glutathionylated by exercise and beta_2_-adrenergic stimulation. Our data suggest that glutathionylation contributes to the complex regulation of Na,K-ATPase function in human skeletal muscle. Glutathionylation of the Na,K-ATPase may explain reductions in maximal Na,K-ATPase activity after exercise, which may be involved in muscle fatigue.

## Introduction

Ion gradients across the muscle membrane undergo pronounced perturbations during intense muscle contractions. These activity-induced changes in ion distribution affect muscle excitability and may lead to impairment of force development (muscle fatigue). The Na,K-ATPase (=Na,K-pump) counteracts the rundown of transmembrane gradients of Na^+^ and K^+^. Regulation of the Na,K-ATPase is therefore important for muscle functioning. It is generally accepted that the Na,K-ATPase is upregulated during muscle activity by a multifactorial process that includes sensitivity to hormones and elevated intracellular Na^+^ concentrations. Moreover, purinergic stimulation may be involved (Walas and Juel 2012; Juel et al. 2014).

Reactive oxygen species are generated in skeletal muscles during activity (Reid 2001; Bailey et al. 2003; Nyberg et al. 2014), which may lead to chemical modification of muscle proteins of importance for muscle function. The oxidative modifications involve formation of disulphide bonds between glutathione and reactive cysteine thiols (S-glutathionylation). It has been reported that oxidative stress (glutathionylation) of the Na,K-ATPase proteins may lead to modifications in Na,K-ATPase function in myocardial (Shattock and Matsuura 1993; Figtree et al. 2009; Liu et al. 2013) and rat skeletal muscle (Juel 2014). Both the *α* and *β*1 subunits along with the regulatory subunit phospholemman (FXYD) of the Na,K-ATPase have been shown to be subjected to glutathionylation (Figtree et al. 2009; Bibert et al. 2011; Liu et al. 2012; Petrushanko et al. 2012; Xianya et al. 2014). In a recent study, treatment with oxidized glutathione (GSSG) was also shown to increase glutathionylation and reduce maximal in vitro Na,K-ATPase activity in a dose dependent manner and with a higher effect in membranes from oxidative compared to glycolytic rat muscles (Juel 2014).

A number of studies have shown that the Na,K-ATPase activity (quantified with the 3-*O*-MFPase method) in humans is reduced after muscle activity. The reduction is minor (5%) after short intense exercise of approximately 5 min duration, but up to 30% after prolonged (30 min) exercise (McKenna et al. 2008). In contrast, we have shown that the Na,K-ATPase activity (quantified with an ATPase assay) is upregulated after short (∼4 min) intense muscle activity in humans (Juel et al. 2013). However, in a recent study with more prolonged exercise (10 min warm-up, three 4-min bouts at 75% of 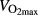 and one bout at 120% 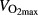 to exhaustion) the maximal in vitro ATPase activity was reduced (Hostrup et al. 2014), which may be explained by glutathionylation. It is therefore hypothesized that glutathionylation reduces Na,K-ATPase activity in skeletal muscle. The first aim of the present study was to investigate if prolonged muscle activity in humans is associated with changes in glutathionylation of Na,K-ATPase protein isoforms, which could explain the contradictory results observed in previous studies. Furthermore, it has been suggested that beta_2_-adrenergic stimulation induces protein kinase A (PKA) mediated glutathionylation of the Na,K-ATPase proteins in heart muscle (Galougahi et al. 2013). In a recent study, beta_2_-adrenergic stimulation with terbutaline counteracted the exercise-induced reduction in Na,K-ATPase activity, whereas the expected terbutaline-induced increase in Na,K-ATPase activity of nonfatigued skeletal muscle was absent (Hostrup et al. 2014). Therefore, in order to determine if glutathionylation could be an underlying mechanism for changes in Na,K-ATPase activity, the second aim was to study the effect of PKA stimulation with terbutaline on glutathionylation in human skeletal muscle. For these purposes, we used human muscle samples that were obtained for a previous study (Hostrup et al. 2014).

## Materials and Methods

### Ethical approval

The study was performed in accordance with the Helsinki II declaration and was approved by the local ethics committee of the city of Copenhagen (H-1-2011-080). The details of the study have been published before (Hostrup et al. 2014). In short: muscle biopsies were obtained from vastus lateralis before (Control samples) and after cycle ergometer exercise (Exercise samples). In addition, experiments were carried out with and without beta_2_-adrenergic stimulation with the selective beta_2_-adrenoceptor agonist terbutaline (20 mg; Bricanyl Turbohaler, AstraZeneca, Sweden). The resulting serum terbutaline concentration at the time of exhaustion was 22 ng mL^−1^ (0.1 μmol L^−1^). In the terbutaline experiments, muscle samples were obtained at rest (Terbutaline samples) and after exercise (Terbutaline plus Exercise samples). Thus, four muscle samples were obtained from each subject. The cycle ergometer exercise included 10 min warm-up at 150 W followed by three 4-min exercise bouts at an intensity corresponding to 75% of subjects’ maximal oxygen uptake (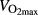), each interspersed by 4 min recovery. Immediately following the third bout, load was increased to 120% of 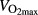 that subjects performed to exhaustion (∼130 sec). In the main experiments the “Control” samples were obtained at rest, but after low-intensity warm-up. To ensure correct quantification of the glutathionylation at rest, we therefore included 10 samples from another group of subject. These samples (“rest-samples”) were obtained before warm-up.

### Treatment of samples

Muscle samples were immediately frozen in liquid nitrogen and stored at −80°C until they were used. Muscle were homogenized for 30 sec (Polytron PT 2100) in 250 mmol L^−1^ mannitol, 30 mmol L^−1^ l-histidine, 5 mmol L^−1^ EGTA, and 0.1% deoxycholate, adjusted to pH 6.8 with Tris-base. This homogenate was used for immunoprecipitation and the subsequent Western blotting. Part of the homogenate was centrifuged at 3000× ***g*** for 30 min, and the resulting supernatant was centrifuged at 190,000× ***g*** for 90 min (at 4°C). The final pellets (called the 190,000× ***g*** fraction) were used for the Na,K-ATPase assay. The protein contents of samples were determined in triplicate using a bovine serum albumin standard (DC protein assay; Bio-Rad, Richmond, CA).

### Na,K-ATPase assay

Na^+^-stimulated Na,K-ATPase activity was determined by measuring ATP hydrolysis. Released inorganic phosphate (P_i_) was detected using a malachite-based Biomol Green reagent (Biomol AK-111; Enzo Life Sciences, Farmingdale, NY) as previously described (Juel et al. 2013). Samples (2 *μ*g protein) were suspended in 70 *μ*L assay buffer (10 mmol L^−1^ KCl, 5 mmol L^−1^ MgCl_2_, 50 mmol L^−1^ Tris-base, 5 mmol L^−1^ EGTA, pH 7.4). Na^+^ was added to the samples to a final concentration of 0, 2, 4, 6, 10, 20, 40, or 80 mmol L^−1^ (the ionic strength was kept constant by substituting NaCl with choline chloride). After 5 min of preincubation at 37°C, the reaction was started by adding Mg-ATP to a final concentration of 0.5 mmol L^−1^. After 30 min, the reaction was terminated by adding 1 mL Biomol Green reagent at room temperature. After 30 min incubation, absorbance was read at 620 nm and [P_i_] was calculated from a standard curve. All samples were run in duplicate (activity at 0 mmol L^−1^ Na^+^ was measured four times), and the ATPase activity at 0 mmol L^−1^ Na^+^ was subtracted from all of the activity values. The Na,K-ATPase assay could only be applied to the 190,000× ***g*** fraction, due to the inevitable high background Ca^++^-ATPase activity in unpurified samples.

### 3-O-MFPase activity

The 3-*O*-MFPase activity in muscle homogenates was measured as previously described (Juel et al. 2013). Briefly, samples (10 *μ*g protein) were incubated in assay medium (5 mmol L^−1^ MgCl_2_, 1.25 mmol L^−1^ EDTA, 1.25 mmol L^−1^ EGTA, 100 mmol L^−1^ Tris base, pH 7.4) for 1 min before adding 3-*O*-methylfluorescein phosphate (3-*O*-MFP) to a final concentration of 160 μmol L^−1^. After 90 sec, KCl was added to a final concentration of 10 mmol L^−1^ to stimulate the 3-*O*-MFP reaction, which was monitored continuously for the next 120 sec. All measurements were done with stirring at 37°C using a spectrofluorometer (Ex 475 nm, Em 515 nm) with a continuous recording facility. The slopes of the curves were calculated in an Excel worksheet.

### In vitro glutathionylation

Muscle samples (190,000 ***g*** fraction, control samples) were incubated in oxidized glutathione (GSSG; Sigma-Aldrich G4626, St. Louis, MO) for 20 min at 37°C and the Na,K-ATPase activity measured in control and GSSG-treated muscle as described above.

### Quantification of glutathionylation

Western blotting of homogenized muscle material has shown that many proteins are susceptible to glutathionylation (Mollica et al. 2012). To study the glutathionylation of Na,K-ATPase subunits it is therefore necessary to use a purification step to isolate the subunits. The level of glutathionylation was studied with two independent techniques.

#### Method 1

Immunoprecipitation with Na,K-ATPase *α* and *β* subunit antibodies. The immunoprecipitate was divided in two parts and used in Western blots both for quantification of *α* and *β* subunits with other antibodies and for quantification of glutathionylation with the anti-GSH antibody (MAB5310) (and a sample buffer without the reducing agent dithiothreitol, DTT). The (relative) glutathionylation was calculated as the ratio between the labeling with anti-GSH and the *α* and *β* subunit isoform labeling. The glutathionylation of *α* and *β* subunit in the homogenate could not be measured with this method due to the presence of other glutathionylated proteins, the yield of glutathionylated *α* and *β* subunit proteins could therefore not be calculated.

#### Method 2

Glutathionylated proteins were immunoprecipitated with the anti-GSH antibody (#101-A; Virogen, Watertown, MA) and afterwards samples were subjected to Western blotting and Na,K-ATPase isoforms were detected with *α* and *β* subunit antibodies (sample buffer including DTT). The glutathionylation of *α* and *β* subunits was evaluated by calculating the ratio between isoform labeling after immunoblotting (with anti GSH) and the total labeling in the homogenate.

### Immunoprecipitation

The muscle homogenate (100 or 200 *μ*g of protein) was incubated in an ice-cold lysis buffer (100 mmol L^−1^ NaCl, 20 mmol L^−1^ Tris-base, 10 mmol L^−1^ NaF, 1 mmol L^−1^ PMSP, 1 mg mL^−1^ of the detergent C_12_E_8_, pH 7.4) for 2 h. The appropriate antibody was added, incubated over night at 5°C with end-over-end rotation, succeeded by spinning at 20,000× ***g*** for 20 min to remove the nonlysed fraction. The supernatant was mixed with 15 *μ*L of packed protein G agarose beads (#16-266; Millipore, Darmstadt, Germany). After 4 h of incubation, the beads were sedimented and washed with a lysis buffer (five times). A sample buffer was added, and the mixture was heated to 56°C for 20 min, and sedimented, and the supernatant was used for immunoblotting.

### Western blotting

The samples were mixed with a sample buffer (2 mmol L^−1^ Tris-HCl, 0.2 mmol L^−1^ EDTA, 20 mmol L^−1^ DTT, 4% SDS, 10% glycerol, 0.04% bromophenol blue, pH 8.0). Equal amounts of protein were loaded into each lane and separated by 8–18% SDS–PAGE (Excel 8–18% gradient gel, Amersham).The proteins were then electroblotted onto a polyvinylidene difluoride membrane (Immobilon-P; Millipore). The membrane was blocked for 60 min at room temperature in a TS-buffer (10 mmol L^−1^ Tris-Base, 0.9% NaCl, pH 7.4) containing 2% BSA, 1% skimmed-milk powder, and 0.1% Tween-20 before incubation with a primary antibody diluted in a similar buffer overnight (4°C). After treatment with a horseradish-peroxidase-coupled secondary antibody (Dako, Copenhagen, Denmark) for 90 min at room temperature, the membrane was repeatedly washed in TS-buffer with or without 0.05% Tween-20. The membrane was incubated with enhanced chemiluminescence reagent (ECL; Amersham) and visualized in an ImageQuant LAS 4000 image station. The samples that were to be compared were loaded on the same gel. The relative protein concentrations were quantified by the image station software.

### Antibodies

Glutathionylation was detected with the anti-glutathione antibody MAB5310 (Millipore). The immunoprecipitation of the glutathionylated proteins was carried out with a monoclonal anti-glutathioine (GSH) antibody (#101-A; Virogen). The Na,K-ATPase *α*1 isoform was immunoprecipitated with SC-21712 antibody (Santa Cruz Biotech., Dallas, TX) and immunedetected with the sc-28800 antibody (H-300; Santa Cruz Biotech.). All *α* subunits (*α*(all)) were immunoprecipitated with sc-28800 and immunodetected with the *α*5 antibody (Developmental Studies Hybridoma Bank, University of Iowa, Iowa City).

The *β*1 isoform was immunodetected with a polyclonal antibody generously provided by Dr. P.A Pedersen, University of Copenhagen. The *β*2 isoform was detected with the polyclonal antibody 06-1711 (Millipore).

### Statistics

**Figure 1:**
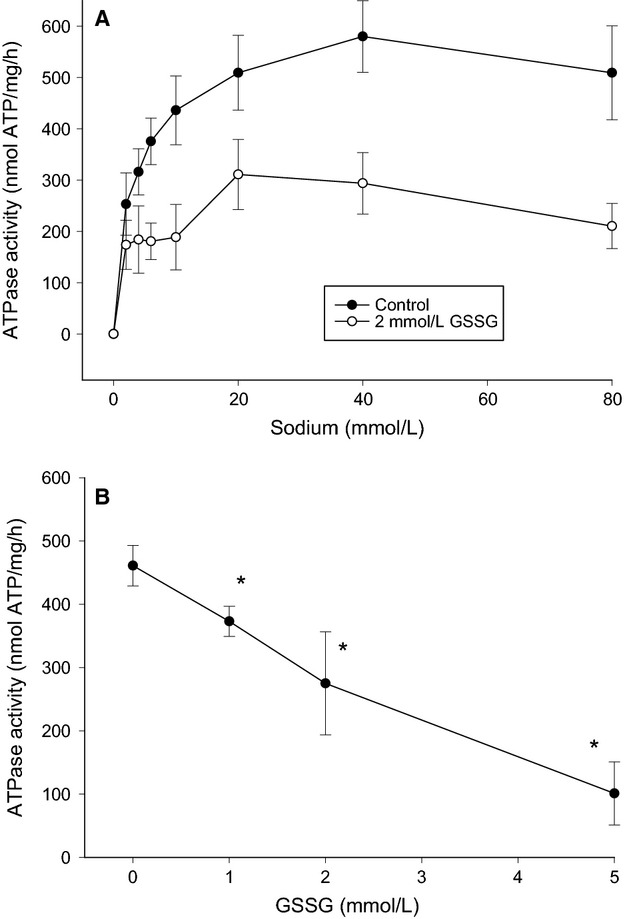
In vitro glutathionylation and Na,K-ATPase activity. (A) Na^+^ dependent Na,K-ATPase activity with and without preincubation with 2 mmol L^−1^ GSSG (oxidized glutathione). Data from six subjects, mean ± SE shown. The two curves are significantly different (two-way ANOVA). (B) Dose–response curve for the effect of GSSG on *V*_max_ of Na^+^ dependent Na,K-ATPase activity. *V*_max_ was obtained from a curve fit (Hill equation) to the mean Na^+^ dependent ATPase activity in the four group of experiments (*n* = 18 for control (without GSSG) and *n* = 6 for 1, 2 and 5 mmol L^−1^ GSSG, ±SD obtained from the curve fit). *Significantly different (*P* < 0.05) from control (without GSSG).

**Figure 2:**
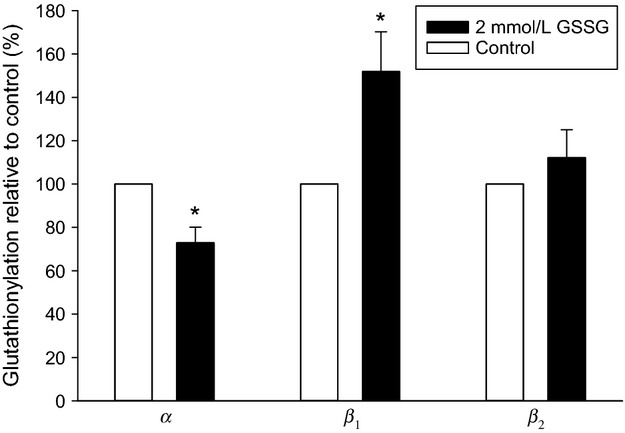
In vitro glutathionylation with GSSG. Muscle homogenates (only control samples) were treated with 2 mmol L^−1^ GSSG for 20 min before immunoprecipitation with an anti-GSH antibody. The amounts of *α*, *β*1, and *β*2 proteins in the immunoprecipitate were quantified with Western blotting. The glutathionylation was calculated relative to the untreated sample for each isoform (*n* = 10, ±SE shown). *Significantly different from untreated sample (*P* < 0.05).

**Figure 3:**
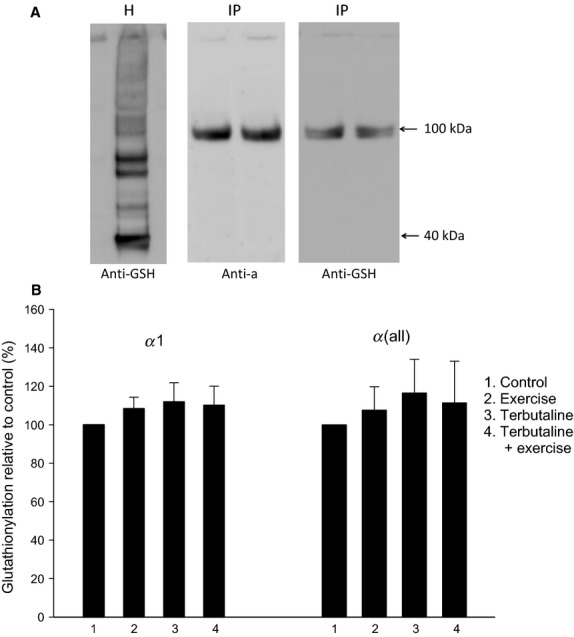
The relative level of glutathionylated Na,K-ATPase *α* subunits (Method 1). (A) H; Western blot of muscle homogenate (10 *μ*g protein per lane) labeled with the anti-GSH antibody. IP left: Western blots of two different immunoprecipitates (from 200 *μ*g proteins) using the anti-*α*(all) antibody (H-300) and labeled on the gel with the anti-*α*(all) antibody *α*5. IP right: Western blots of the same two immunoprecipitates labeled with the anti-GSH antibody. (B) Glutathionylation of *α*1 and *α*(all) subunits calculated relative to controls. Mean ± SE (*n* = 10). None of the mean values for exercise, terbutaline, and terbutaline plus exercise were significantly different from the controls.

**Figure 4:**
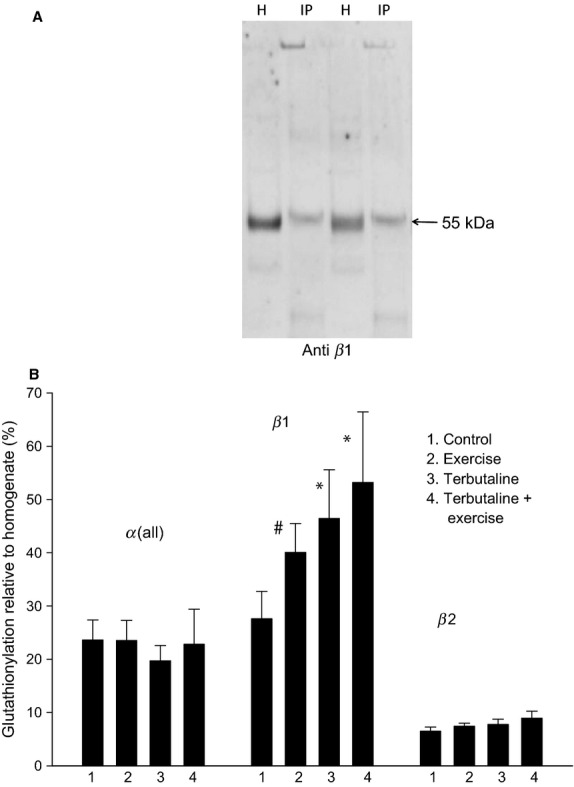
The level of *α* and *β* subunits glutathionylation, the effects of exercise and terbutaline. The glutathionylated proteins were isolated with immunoprecipitation using anti-glutathione antibodies and Na,K-ATPase subunits quantified with Western blots of the immunoprecipitate (Method 2). (A) H; examples of the *β*1 subunits detected in the Western blots of two different homogenates (10 *μ*g protein in each lane). IP: *β*1 subunits detected with anti-*β*1 antibodies in immunoprecipitates (from100 *μ*g protein) obtained from the same two homogenates using the anti-GSH antibody. (B) The absolute level of glutathionylated *α* and *β* subunits. The values are calculated relative to the total density of *α*(all), *β*1, and *β*2 proteins, respectively, in the homogenates used for immunoprecipitation. Mean ± SE. *Significantly different from the controls (*P* < 0.05). #*P* < 0.1.

The effect of GSSG treatment on Na,K-ATPase activity was analyzed with analysis of variance (two-way ANOVA) (Fig.1A). *V*_max_ for Na^+^ stimulated ATPase activity (Fig.1B) was determined for each group of experiments by nonlinear regression (Sigma Plot software, San Jose, CA) with a Hill equation. Data are presented as mean ± SEM. The data obtained from Western blotting (Figs. 2–4) were compared with Student′s paired *t*-test. *P* < 0.05 was considered significant. Muscle samples were obtained from 10 subjects. The in vitro glutathionylation experiments (Fig.1) were carried out with a reduced number of samples due to lack of material, whereas the immunoprecipitation experiments (Figs. 2–4) contain material from all subjects.

## Results

### Effect of in vitro glutathionylation

Twenty minute preincubation with oxidized glutathione (GSSG) was used to induce in vitro glutathionylation in purified muscle membranes and, subsequently, the Na,K-ATPase activity was quantified with the ATPase assay. Figure1A shows the effect of 2 mmol L^−1^ GSSG on Na^+^ dependent Na,K-ATPase activity. It can be seen that 2 mmol L^−1^ GSSG significantly reduced (*P* < 0.05) the Na,K-ATPase activity. The effect of 1, 2 and 5 mmol L^−1^ GSSG on mean *V*_max_ (obtained from a curve fit with a Hill equation) is depicted in Figure1B. GSSG inhibited the maximal in vitro Na,K-ATPase activity in a dose-dependent manner.

### Quantification of GSSG-induced in vitro glutathionylation

Muscle homogenates were preincubated with 2 mmol L^−1^ GSSG. Samples were subsequently immunoprecipitated with an anti GSH antibody and the Na,K-ATPase isoforms of the immunoprecipitate were then quantified with Western blotting. The level of glutathionylation was calculated relative to untreated control samples (Fig.2). The relative glutathionylation of the *α* isoforms was reduced by 27% (*P* < 0.05), the relative level of glutathionylated *β*1 isoforms was increased by 52% (*P* < 0.05), whereas the relative level of *β*2 isoforms remained unchanged.

### 3-O-MFPase measurements

The preincubation of muscle homogenates with 2 mmol L^−1^ GSSG reduced the Na,K-ATPase activity measured with the 3-*O*-MFPase technique to 42 ± 19% of the untreated controls (*n* = 6, *P* < 0.05).

### Relative glutathionylation of α subunits, and the effect of exercise and terbutaline

Testing muscle homogenates with anti-GSH antibodies resulted in a number of labeled proteins (Fig.3), which cannot be immediately identified. A purification (immunoprecipitation) step was therefore necessary to identify the glutathionylated Na,K-ATPase *α* subunits (Method 1). The immunoprecipitation with Na,K-ATPase *α* subunit antibodies resulted in the purification of one specific protein band at 100 kDa seen on the Western blot (Fig.3A). Another part of the immunoprecipitate was ran on a similar gel and tested with anti-GSH antibodies. Only one band at the same molecular weight was labeled. The relative level of glutathionylation was evaluated by the glutathionylation/density ratio of the subunit proteins quantified on two different gels. The glutathionylation levels for *α*1 and *α*(all) were not different in the four groups of human samples (Fig.3B).

Similar measurements could not be carried out for the *β* subunits. Apparently, the *β* subunit proteins stick to other proteins because of the nonreducing buffer.

There was an inverse correlation between the level of *α*(all) glutathionylation as quantified in the present study and of the in vitro *V*_max_ of the Na,K-ATPase measured in the same muscle samples (Hostrup et al. 2014) (*n* = 40, *P* < 0.01), but with a low coefficient of determination (*R*^2^ = 0.16). A similar analysis for the *β* subunits could not be performed, since the total amount of *β* subunits were not measured, and the ratio between *β*1 and *β*2 is unknown and probably different for each sample.

### Glutathionylation of α and β subunits

To access the absolute level of glutathionylation of the *α*(all) and *β* isoforms Method 2 was used: first we isolated the glutathionylated proteins by immunoprecipitation with an anti-GSH antibodies, than we identified the Na,K-ATPase proteins in the immunoprecipitate with Western blotting. The relative glutathionylation could then be calculated from isoform content in the immunoprecipitate and in the untreated muscle homogenate (Fig.4A). For the control samples, the density of glutathionylated *β*1 was approximately 28% of the density of the total *β*1 in the homogenate used for immunoprecipitation. In the supplementary experiments *β*1 glutathionylation in the “rest” samples was 25.6 ± 9% (*n* = 10, not different from the Control samples). The density of glutathionylated *β*1(40%) tended to be higher in the control samples after exercise (*P* < 0.1). The density of glutathionylated *β*1 was higher (46%) (*P* < 0.05) with terbutaline than it was with the control (Fig.4B). Thus, terbutaline increased glutathionylation in nonfatigues muscle. Terbutaline plus exercise further increased glutathionylation to 53% of control (*P* < 0.05). Similar experiments with *β*2 antibodies detected 6–9% glutathionylation and no effects of exercise and terbutaline (Fig.4B). Similar experiments with *α*(all) antibodies detected 23% glutathionylation in the control samples and no significant effects of exercise and terbutaline plus exercise.

## Discussion

This is the first study to investigate the effect of glutathionylation on Na,K-ATPase activity in human skeletal muscle. The major findings of the present study are (1) that in vitro glutathionylation reduces the maximal Na,K-ATPase activity, (2) that a basal glutathionylation is present in samples taken at rest, and (3) that exercise and beta_2_-adrenergic stimulation increase the glutathionylation of the Na,K-ATPase *β*1 subunit, whereas the glutathionylation level is constant for the other subunits. Thus, the study supports a role for glutathionylation in skeletal muscle Na,K-ATPase regulation.

### In vitro glutathionylation

The in vitro incubation with GSSG (oxidized glutathione) demonstrated a dose-dependent reduction in maximal the Na,K-ATPase activity in human skeletal muscle samples (Fig.1B). Immunoprecipitation experiments revealed that the in vitro GSSG incubation was associated with an increased level of Na,K-ATPase *β*1 subunit glutathionylation (Fig.2). Together, these findings suggest a causative invers correlation between glutathionylation level and maximal Na,K-ATPase activity of skeletal muscle in humans. These observations are consistent with the glutathionylation-dependent inhibition shown in rat muscle (Juel 2014) and cardiac myocytes (Liu et al. 2013).

In Figure2, it can also be seen that the GSSG treatment induced an apparent reduction in *α* subunit glutathionylation. However, it has been reported that increased glutathionylation is associated with a decreased *α*/*β* subunit co-immunoprecipitation (Figtree et al. 2009). Thus, it appears that some co-immunoprecipitation is present in these types of experiments. This phenomenon is discussed below.

### Basal level of glutathionylation

In the present study, the level of glutathionylation of the human Na,K-ATPase *α* subunits in samples obtained at rest was accessed with two methods. While the first method (Method 1) did not allow calculation of the absolute level of glutathionylation of the *α* subunits, some glutathionylation was detectable (Fig.3). The second method (Method 2) revealed approximately 20% glutathionylation of the *α* subunits (Fig.4).

There appears to be some controversy in the literature regarding *α* subunit glutathionylation. Some studies report redox sensitivity of the Na,K-ATPase *α* subunit in myocardial tissue (Petrushanko et al. 2012; Xianya et al. 2014), whereas other studies found no evidence for the existence of glutathionylated *α*1 subunits (Figtree et al. 2009; Liu et al. 2012). The present study (Fig.3) supports that *α* subunits can be glutathionylated in human muscles. In addition, membranes of glycolytic rat muscle, that contain low levels of the glutathione sensitive *β*1 isoforms (Juel 2009), the Na,K-ATPase activity was sensitive to oxidized glutathione (GSSG), which suggests that other protein isoforms could be affected. Taken together these findings suggest that *α* subunits are subjected to glutathionylation. These experiments also found no differences between specific anti-*α*1 and unspecific anti-*α*(all) antibodies, which indicates that the glutathionylation is similar between *α* isoforms.

Approximately 28% of the *β*1 subunits were glutathionylated in the control samples (Fig.4). The observation of a basal level of glutathionylated Na,K-ATPase subunits is in accordance with the presence of oxidized glutathione (GSSG) in resting muscle. The levels of GSSG and GSH are approximately 0.1 and 0.5 mmol L^−1^, respectively, in human skeletal muscle at rest (Medved et al. 2004; Trewin et al. 2015), and the GSSG concentration in venous blood is 0.11 mmol L^−1^ at rest (Nyberg et al. 2014). Thus, the in vivo concentrations are lower that the concentrations used in the present in vitro experiments.

### Effect of exercise and terbutaline

We also compared the levels of glutathionylation in muscle samples obtained before and after approximately 30 min of intermittent muscle activity including a 10-min warm-up period. While it is generally accepted that cAMP dependent PKA stimulation acutely increases the Na,K-ATPase activity in rat muscle (Clausen and Flatman 1977), it has also been reported that PKA increases glutathionylation of Na,K-ATPase subunits in cardiac myocytes, which may inhibit Na,K-ATPase activity (White et al. 2010; Galougahi et al. 2013). We therefore included muscle samples obtained from human subjects treated with the beta_2_-adrenoceptor agonist terbutaline known to induce cAMP and PKA activity (Hostrup et al. 2014).

We observed that human Na,K-ATPase *β*1 subunits were subjected to changes in the degree of glutathionylation with exercise and terbutaline, whereas no changes were observed for the other subunits (Fig.4). During exercise, terbutaline and terbutaline plus exercise, the glutathionylation of the *β*1 subunits increased to 32%, 38% and 45%, respectively, relative to control (Fig.4).

The maximal in vitro Na,K-ATPase activity (*V*_max_) of the control samples used in the present study was shown by Hostrup et al. (2014) to be 388 ± 33 nmol ATP h^−1^ mg^−1^ of protein in control samples and to be reduced to 293 ± 49 nmol ATP h^−1^ mg^−1^ of protein after exercise (*n* = 10, *P* < 0.05). With terbutaline, the corresponding values were 343 ± 43 and 354 ± 20 nmol ATP h^−1^ mg^−1^ of protein before and after exercise, respectively (not significantly different) (Hostrup et al. 2014).

It is therefore suggested that the changes in in vitro Na,K-ATPase activity in skeletal muscle are influenced by glutathionylation of subunits. This is also supported by studies in heart muscle, in which the glutathionylation of the *β*1 subunit reduces Na,K-ATPase activity (Figtree et al. 2009). Therefore, the glutathionylation of the *β*1 subunits (Fig.4) is the most likely candidate for the reduced Na,K-ATPase activity with exercise and for the surprising lack of stimulatory effect of terbutaline. It must, however, be noted that the highest level of *β*1 glutathionylation was not associated with the lowest Na,K-ATPase activity. For instance, the Na,K-ATPase activity with terbutaline was higher (Hostrup et al. 2014) than in control, although terbutaline induced glutathionylation. We have previously shown (Hostrup et al. 2014) that exercise and terbutaline increased PLM^Ser68^ phosphorylation, which is known to increase the affinity for Na^+^ of the Na,K-ATPase activity. Although an increased affinity (lower K_m_) is expected to mainly increase Na,K-ATPase activity at low Na^+^ concentrations, PKC activation and PLM phosphorylation have been demonstrated to affect *V*_max_ (Juel et al. 2014). This mechanism may in part counteract the negative effect of *β*1 subunit glutathionylation.

The difference between the degree of glutathionylation in *β*1 and *β*2 subunits is probably related to the presence of a reactive cysteine (Cys46) in the *β*1 isoform (Figtree et al. 2009). This fits with the finding of nearly double sensitivity to oxidized glutathione in oxidative fibers (mainly *β*1 subunits) compared to glycolytic (mainly *β*2 subunits) of rat muscles (Juel 2014).

The *β*1 subunit distribution in human skeletal muscle is not clearly related to fiber type (Thomassen et al. 2013; C. Juel, unpublished observation) and the inhibitory effect of glutathionylation may therefore affect all fiber types.

### Evaluation of methods

In the immunoprecipitation experiments, the possibility of co-immunoprecipitation must be considered. In Method 1 the immunoprecipitation was carried out with Na,K-ATPase *α* isoform antibodies and the immunoprecipitate was then subjected to Western blotting. Subsequently, the level of glutathionylation was quantified from an anti-GSH antibody response at the molecular weight of the *α* isoform. Glutathionylated proteins were not seen at other molecular weights (Fig.3A). Therefore, it is unlikely that *α*/*β* isoform co-immunoprecipitation influenced the results obtained in Method 1.

In Method 2 the immunoprecipitation was carried out with an anti-GSH antibody and subsequently the amounts of *α* and *β* subunits were quantified in the immunoprecipitate with Western blotting. The apparently reduced glutathionylation of the *α* subunits shown in Figure2 could be due to a reduced *α*/*β* co-immunoprecipitation, as co-immunoprecipitation is known to be reduced with glutathionylation (Figtree et al. 2009). However, in Figure4B the increased glutathionylation of the *β*1 subunit with exercise and terbutaline was not associated with changes in the level of glutathionylation of the *α* subunits. Since the lysing procedure and immunoprecipitation was the same in the two experiments (Figs.2 and 4), it is therefore unlikely that the increased *β*1 subunit glutathionylation with exercise and terbutaline was due to changes in co-immunoprecipitation.

Approximately 28% of the *β*1 subunits were glutathionylated in the control samples (Fig.4), whereas the *β*2 subunit showed approximately 8% glutathionylation. The latter value could be an artefact due to co-immunoprecipitation. A similar co-immunoprecipitation could be assumed for the *β*1 subunit. Consequently, the most likely specific glutathionylation of the *β*1 isoform is approximately 20%.

### The effect of glutathionylation on ATPase function

Skeletal muscle Na,K-ATPase activity quantified with the 3-*O*-MFPase technique has been demonstrated to decrease with exercise; a phenomenon called Na,K-pump inactivation (McKenna et al. 2008). A number of studies have shown that short lasting intense exercise has a moderate inhibitory effect on the maximal in vitro Na-K-ATPase activity, and that prolonged exercise (20–30 min) reduced the activity by up to 30%. In contrast, the maximal in vitro Na,K-ATPase activity has been demonstrated to increase with short intense exercise when activity was quantified with a more direct ATPase assay (Juel et al. 2013). This difference could be related to the quantification methods used. We have reported that the ATPase assay is sensitive to acute phosphorylation-dependent increases in the Na,K-ATPase Na^+^ affinity, whereas the 3-*O*-MFPase technique is not sensitive to such changes (Juel et al. 2013). In addition, we have previously shown that the ATPase assay is sensitive to glutathionylation-induced reductions in Na,K-ATPase activity (Juel 2014). The present study demonstrated that the 3-O-MFPase technique is also sensitive to glutathionylation-induced changes. Taken together, the different properties of the methods used explain why short high intensity exercise is reported to increase the in vitro maximal Na,K-ATPase activity in some studies and to decrease the activity in other studies. With prolonged exercise a decrease in the maximal Na,K-ATPase activity has been seen with both techniques. Thus, it appears that the negative effects overrule the positive during prolonged exercise.

Of interest, an infusion of the antioxidant N-acetylcysteine in human subjects has been demonstrated to partly attenuate the decline in muscle in vitro Na,K-ATPase activity (3-*O*-MFPase activity) and to delay fatigue during prolonged exercise (McKenna et al. 2006). Since *N*-acetylcysteine is reported to increase muscle GSH/GSSG ratio (Medved et al. 2004; Trewin et al. 2015) and thereby probably decrease glutathionylation of Na,K-ATPase subunits, these findings support the involvement of glutathionylation in exercise-induced changes in muscle Na,K-ATPase activity. However, other mechanisms involving glutathionylation may also be of importance. In vitro stimulation of protein kinases (PKA and PKC) has been shown to acutely increase Na,K-ATPase activity (Na^+^ affinity) in purified rat and human muscle membranes (Walas and Juel 2012; Juel et al. 2014). However, other studies have observed that glutathionylation of PKA and PKC can inhibit the activity of these kinases and might reduce Na,K-ATPase activity probably by reducing the phosphorylation state of the regulatory unit FXYD (Liu et al. 2013) or by an effect on *β* subunit glutathionylation (White et al. 2010). Furthermore, studies also suggest that FXYD glutathionylation can counteract the inhibitory effect of *β* subunit glutathionylation on Na,K-ATPase activity (Bibert et al. 2011). The effect of glutathionylation on Na,K-ATPase function is therefore complex.

In conclusion, the present study supports that Na,K-ATPase subunits are subjected to a basal level of glutathionylation in skeletal muscle of humans. In many studies, the maximal Na,K-ATPase activity is evaluated from the relative amount of Na,K-ATPase subunits measured by Western blotting or the total amount of Na,K-pumps determined with ouabain labeling. The finding of a basal glutathionylation indicates that the maximal functional Na,K-ATPase activity is lower than the theoretical maximal value. In addition, the present study showed that glutathionylation contributes to the complex regulation of the Na,K-ATPase activity during exercise and beta_2_ adrenergic stimulation in skeletal muscles of humans. Exercise-induced glutathionylation is likely one of the underlying mechanisms involved in muscle fatigue.
